# Improving health equity through health care systems research

**DOI:** 10.1111/1475-6773.14192

**Published:** 2023-11-28

**Authors:** Deena J. Chisolm, Jerome A. Dugan, Jose F. Figueroa, Meghan B. Lane‐Fall, Dylan H. Roby, Hector P. Rodriguez, Alexander N. Ortega

**Affiliations:** ^1^ Abigail Wexner Research Institute, Nationwide Children's Hospital, Department of Pediatrics The Ohio State University College of Medicine Columbus Ohio USA; ^2^ Department of Health Systems and Population Health, School of Public Health University of Washington Seattle Washington USA; ^3^ Harvard T.H. Chan School of Public Health Brigham and Women's Hospital Cambridge Massachusetts USA; ^4^ Department of Anesthesiology and Critical Care Medicine, Perelman School of Medicine and Leonard Davis Institute of Health Economics University of Pennsylvania Philadelphia Pennsylvania USA; ^5^ Department of Health, Society, and Behavior, Program in Public Health University of California, Irvine Irvine California USA; ^6^ Division of Health Policy and Management, School of Public Health University of California, Berkeley Berkeley California USA; ^7^ Department of Health Management and Policy, Dornsife School of Public Health Drexel University Philadelphia Pennsylvania USA; ^8^ Present address: Thompson School of Social Work & Public Health University of Hawaii at Manoa Honolulu Hawaii USA

**Keywords:** health disparities, health equity, health services research, health care delivery systems, social determinants of health

## Abstract

**Objective:**

To describe health equity research priorities for health care delivery systems and delineate a research and action agenda that generates evidence‐based solutions to persistent racial and ethnic inequities in health outcomes.

**Data Sources and Study Setting:**

This project was conducted as a component of the Agency for Healthcare Research and Quality's (AHRQ) stakeholder engaged process to develop an *Equity Agenda and Action Plan* to guide priority setting to advance health equity. Recommendations were developed and refined based on expert input, evidence review, and stakeholder engagement. Participating stakeholders included experts from academia, health care organizations, industry, and government.

**Study Design:**

Expert group consensus, informed by stakeholder engagement and targeted evidence review.

**Data Collection/Extraction Methods:**

Priority themes were derived iteratively through (1) brainstorming and idea reduction, (2) targeted evidence review of candidate themes, (3) determination of preliminary themes; (4) input on preliminary themes from stakeholders attending AHRQ's 2022 Health Equity Summit; and (5) and refinement of themes based on that input. The final set of research and action recommendations was determined by authors' consensus.

**Principal Findings:**

Health care delivery systems have contributed to racial and ethnic disparities in health care. High quality research is needed to inform health care delivery systems approaches to undo systemic barriers and inequities. We identified six priority themes for research; (1) institutional leadership, culture, and workforce; (2) data‐driven, culturally tailored care; (3) health equity targeted performance incentives; (4) health equity‐informed approaches to health system consolidation and access; (5) whole person care; (6) and whole community investment. We also suggest cross‐cutting themes regarding research workforce and research timelines.

**Conclusions:**

As the nation's primary health services research agency, AHRQ can advance equitable delivery of health care by funding research and disseminating evidence to help transform the organization and delivery of health care.

**What is known on this topic**

- Racial and ethnic and sociodemographic health care inequities in the United States are pervasive and persist.
- Health care delivery systems have contributed to and maintain these disparities.
- Quality improvement programs and health policy innovations have facilitated modest equity improvements, but research on which approaches works best and how to effectively scale the most promising equity‐focused interventions is limited.

**What this study adds**

- Review of the research evidence and expert consensus identified multiple themes with substantial research gaps for health care delivery system‐based innovations to address health care inequities.
- To be effective, health care policies, programs, and interventions targeting inequities should address the systems of structural and interpersonal discrimination that have created extant inequities.
- The Agency for Healthcare Research and Quality can generate meaningful improvement by supporting the generation of original research, facilitating cross sector scientific collaborations and data sharing, and building the next generation of health equity researchers.

## 1 INTRODUCTION

The landmark publication *Unequal Treatment*, released in 2003, captured the attention of the health care world by formally stating that “*Racial and ethnic disparities in healthcare exist and, because they are associated with worse outcomes in many cases, are unacceptable*.”^1^ The report's findings highlighted the relatively poor quality of care provided to minoritized populations across clinical conditions and health care settings. Today, over 20 years post *Unequal Treatment*, many of these health care inequities persist and continue to contribute to unacceptable inequities in the health of minoritized populations.

Health care delivery systems have been party to creating and maintaining inequities in health care.^2^, ^3^, ^4^, ^5^ Positively, these systems are also increasingly recognizing that they can, and should, be responsible for addressing them.^6^, ^7^, ^8^ Addressing health equity at the health care delivery systems level will require dismantling the structures and behaviors that sustain inequitable health care and advancing new approaches to care that explicitly address the needs of populations that have been historically underserved. It will also require partnerships, collaborations, and investments outside the health care system to impact socioeconomic and structural factors, beyond health care, that drive population health. Importantly, high‐quality research is required to determine the best strategies for success. In 2021, The Agency for Healthcare Research and Quality (AHRQ) launched a multiphase stakeholder engaged process to develop an *Equity Agenda and Action Plan* to guide setting priorities to advance health equity. The Equity and Action Framework that emanated from the first stakeholder meeting, which was held in December of that year, outlined five core research and action themes: healthcare delivery systems; payment; implementation science; social determinants of health; and access to care. This paper discusses priority research themes for advancing health equity through health care delivery systems and offers recommendations for an AHRQ Health Equity Agenda and Action Plan.

## 2 METHODS

### 2.1 AHRQ Equity Agenda and Action Plan

AHRQ convened expert writing teams to conduct evidence‐based narrative reviews of the intersection of health equity and each core research and action theme. The writing teams were asked to identify research gaps that, if filled, could accelerate equity and to make specific recommendations on how AHRQ could serve as a catalyst to drive more equitable health care. For more details on the processes of stakeholder engagement, theme and framework development, and manuscript preparation, see supplemental material and the commentary by Mistry et al. in this issue.^9^

### 2.2 Evidence synthesis

The priority research themes and associated recommendations presented in this study were developed using a stakeholder‐informed expert consensus approach. First, the authors met and generated an extensive list of relevant health care equity themes with research gaps. Group processes were used to reduce and refine the developed list. Once an initial consensus on priority research themes was achieved, targeted literature reviews were conducted for each theme which informed our initial manuscript outline. This outline, including candidate priority themes, was shared with AHRQ and their invited stakeholders in advance of a 2‐day virtual summit on Health Equity held in September of 2022. Participating stakeholders included experts from academia, healthcare, industry, and government. During the summit, stakeholders participated in focused breakout sessions where they discussed the outline and offered insights for content that should be added, expanded, or removed. Following the summit, AHRQ shared recordings from the breakout sessions to inform our manuscript development. Three additional writing meetings were held to integrate the content from the summit with the original outline. A final manuscript proposal which was reviewed by the AHRQ prior to the production of this manuscript.

### 2.3 Guiding conceptual lens

We approached our synthesis of evidence and expertise using a guiding conceptual lens that racism and bias are embedded in the health care delivery system and serve as impediments to achieving health equity. This “structural racism” includes policies and procedures at the organizational, community, and societal levels that constrain health care delivery, reduce access, and influence workforce composition, all resulting in persistent inequities. Discriminatory intent is not needed for such structures to reinforce inequities. Health inequities are also perpetuated by implicit and explicit biases that reduce equitable delivery of high‐quality clinical care and by overt expressions of racism and bias that alienate patients and engender mistrust in the health care delivery system. The importance of anti‐racist and anti‐discriminatory frameworks undergird each health equity research recommendation offered in this study.

## 3 RESULTS

The synthesis of literature, stakeholder input, and expert opinion conducted for this study yielded six priority themes for health care delivery system research and action: (1) institutional leadership, culture, and workforce; (2) data‐driven, culturally tailored care; (3) health equity‐targeted performance incentives; (4) health equity‐informed approaches to health system consolidation and access; (5) whole patient care; (6) and whole community investment. For each theme, we describe the rationale for prioritization and offer recommendations for related future research and action as part of the AHRQ Equity Agenda and Action plan. Recommendations are summarized in Table 1.

**TABLE 1:** Summary of health care delivery systems recommendations for AHRQ's Equity Agenda and Action Plan.

Theme	Research agenda	Action plan
Institutional leadership, culture, and workforce	Best practices in workforce recruitment for diversity Models for integration of diverse lived experience in health care management decision making Health equity outcomes of Diversity, Equity, and Inclusion programs and anti‐burnout initiatives	Publish publicly available white papers and toolkits on evidence‐informed workforce diversity strategies
Data‐driven, culturally tailored care	Best practices for collection of race, ethnicity, and language data Health equity outcomes of culturally tailored care Patient preferences regarding culturally tailored interventions	Share research findings with policy making entities on evidence‐based collection of patient race, ethnicity, and language data by providers and payers Develop toolkits to assist health care organizations maximize completeness and quality of race, ethnicity and language data
Health equity targeted performance incentives	Health equity related effectiveness of performance incentives including different incentive types and incentive recipients Best practices for incentivizing quality and equity in resource‐limited providers	Develop toolkits to assist health care organizations with integrating equity metrics into their performance management systems. Commission the development of equity‐focused evidence‐based quality indicators Utilize federal data to develop publicly available health equity performance data for use in benchmarking.
Health equity‐informed approaches to health system consolidation and access	Impacts of mergers, acquisitions, and consolidation on health equity, health care cost, geographic access and cultural appropriateness of care Implementation research on centering health equity in consolidation and other business decisions Effectiveness of delivery innovation models to provide access in care deserts.	Support the development of geographic information systems to track changes in the health care access, quality, and equity resulting from consolidation and other policy changes.
Whole‐person health	Identify best approaches for payors to fund health‐related social needs interventions delivered by health care systems and by community organizations Quantify the health equity impact of health‐related social needs screening and intervention	Expand access to secondary linked data sets analysis across federal surveys to accelerate research on health outcomes linked to social needs Engage other federal entities responsible for addressing social risk factors such as the Environmental Protection Agency, the Department of Transportation, and the Department of Housing and Urban Development in co‐sponsored research.
Whole Community Investment	Innovative health services research studies at the intersections of race, racism, economic adversity, and other identities in health services research to guide future investments. Effectiveness of existing evidence‐based practices at larger scales	Develop a new health equity research funding models with funding allocated directly to community organizations working in partnership with universities or health care organizations using the Small Business Innovation Research (SBIR) model.
Cross‐cutting Concerns		Leverage existing funding mechanisms (e.g., R25, T32, K‐series, and R01 diversity supplements) to train the emerging health services researchers in methodologically rigorous health equity research Expand project funding periods beyond the traditional 5‐year R01 award

### 3.1 Theme 1: Institutional leadership, culture and workforce

#### 3.1.1 Rationale

Our first theme focuses on understanding how the leadership and culture of organizations can be harnessed to improve equity of care. Much of the success in improving health care quality and safety has been credited to adoption of a “culture of safety” which places safety at the center of every patient encounter, clinical process, and administrative and operational decision.^10^, ^11^ Research is needed to establish the analogous strategies in a “culture of equity” and to test their effectiveness. Example strategies include: commitment to diversity on boards of directors and in executive leadership positions tasked with development of missions, vision, and strategic plans; recruitment of clinicians and staff from populations underrepresented in medicine; and development of health equity knowledge and competencies within staff (e.g., implicit bias, social determinants of health, structural racism, and other forms of discrimination); and implementation of burnout reducing delivery innovations.

#### 3.1.2 Recommendations

An AHRQ equity agenda under this theme must link health services research with health care management research to explore elements of effective executive leadership. Key research questions should identify best practices for recruitment of a diverse leadership team and approaches for integrating those leaders' diverse perspectives and lived experiences into decision making. Research in this space would use implementation science and organizational behavior models to understand predictors of success. AHRQ could also commission case studies of successful and less successful approaches for publication as “white papers.”

Research on approaches for recruitment of diverse clinicians and staff will need to explore how to recruit for diversity within health care fields whose members are not representative of the patients they serve. For example, medicine, nursing, and allied health all have longstanding underrepresentation of Black, Latino, and Indigenous people when compared to U.S. census data. Research of recruiting for diversity will need to consider barriers to entry at every level of training to identify methods to overcome the “pipeline issues” which are often used as an explanation for homogeneous workforces. Research should also further explore how workforce diversity influences patient outcomes and equity gaps. For example, existing research shows that non‐White physicians are more likely to practice in underserved areas^12^, ^13^ and that diverse teams tend to provide more culturally appropriate care with better outcomes.^14^, ^15^ Research should quantify the health equity outcomes of workforce diversity actions to create the business case for continued investment.

Research on the effectiveness of diversity, equity, and inclusion (DEI) programs and diversity training mandates is strongly recommended. Given the significant emphasis being placed on these areas, it is important to know what works^16^, ^17^ AHRQ must also consider questions regarding who is tasked with implementing such programs and how that work is valued. Responsibility for DEI initiatives cannot fall solely in the shoulders of personnel from minoritized populations. When this happens, those tasked with health equity leadership functions risk being “tokenized” and often experience the burden of the “minority tax,” carrying a disproportionate share of the responsibility for equity work.^18^ Research on the personal and professional impact of this burden should also be conducted. AHRQ should publish publicaly available white papers and toolkits on evidence informed workforce diversity strategies derived from research in this area.

Research on burnout, across the health care workforce, should consider its role as a cause of health care inequities. Physician and clinical staff burnout, largely driven by a demanding health care environment with substantial time pressures and emotional intensity,^19^ is characterized by exhaustion, depersonalization, a low feeling of self‐worth, and a negative emotional state. Physicians who self‐report symptoms of burnout may be more prone to explicit and implicit racial biases.^20^ Priority research questions include studies linking burnout and disparities, evaluating novel reimbursement schemes for physicians that reward “equity outcomes” over “volume,” and testing adoption of workplace improvements and digital health technologies that help facilitate care.

### 3.2 Theme 2: Data‐driven, culturally tailored care

#### 3.2.1 Rationale

Culturally tailored care refers to care that meets the specific barriers and preferences of culturally defined groups of individuals and communities. Appropriately designing and offering such care requires the routine collection of race, ethnicity, immigration/citizenship status, and language preference data that enable the disaggregated tracking of quality, access, and patient care experience measures, to understand and meet patients' needs.^21^, ^22^ Presently, quality of demographic data varies by payer and provider. For example, race and ethnicity data collected by Medicaid tends to be less complete than data for individuals enrolled in Medicare.^23^ This difference is likely driven by Medicare's requirement for documentation of race upon enrollment which is optional in Medicaid and varies substantially by state.^24^ Other examples of successful data collection strategies for equity measurement include explaining how the information will be used, using subcategories for individuals who identify as Hispanic/Latino, making responding to the questions mandatory but adding options for “do not know” and “choose not to answer,” and educating health insurance enrollment brokers about the significance of assessing and documenting race, ethnicity, and language information.^25^

Language can be one of the greatest barriers to health care for immigrant populations. Even though health care organizations and payers are legally obligated to provide and pay for interpreter services for patients with limited English language proficiency (LEP), widespread usage of professional interpreters is limited due to high associated costs and the modest strength of research evidence linking interpreter services to improved quality of care.^26^, ^27^ Moreover, research linking professional medical interpreter services with quality of care and patient outcomes has been cross‐sectional in nature, which has limited causal inference and policy impact. There has also been little assessment of the potential for structured quality improvement initiatives to improve the access to and quality of professional interpreter services or culturally tailored interventions.

#### 3.2.2 Recommendations

AHRQ‐supported research identifying the most efficient and effective methods for demographic collection could support evidence‐based best practices for collection of data to inform culturally tailored care. Such research should inform development of toolkits to assist health care organizations in maximizing completeness and quality of race, ethnicity and language data. AHRQ should also share findings with federal entities responsible for policy making regarding evidence‐based data collection expectations for providers and payers. AHRQ should also support research quantifying the effectiveness of culturally tailored care within each subpopulation. For example, to date, no research has examined the impact of introducing new interpreter services for patients or whether that impact differs by language group. Pragmatic clinical trials^28^ and trials designed with the dual goals of assessing implementation and effectiveness^29^ are important future investments that AHRQ should consider making to advance evidence about the effectiveness and costs of interpreter services for patients with LEP and culturally tailored interventions, across minoritized populations, for preventive services, maternal and child health, and chronic disease management. Beyond understanding effectiveness, more evidence is needed regarding patient preferences for a range of “culturally tailored” care models, including patient navigation, peer coaching, and shared decision making with clinicians.

### 3.3 Theme 3: Health equity targeted performance incentives

#### 3.3.1 Rationale

Health care incentives are used as external reinforcements that support motivation toward desired behaviors.^30^, ^31^, ^32^, ^33^, ^34^ Recent attempts to align financial incentives with quality through value‐based purchasing, hospital penalties, accountable care organizations, and the Medicare Quality Payment Program (QPP) have not explicitly targeted the elimination or reduction of health inequities. Instead, physicians, hospitals, and other providers earn bonuses or shared savings by improving their performance on specific metrics that are not directly linked to the health of patients from disadvantaged populations.^35^ For example, the QPP's Merit‐Based Incentive Payments program allows physicians to earn an extra 9% of their submitted Medicare claims as a bonus if they perform well on a set of performance metrics focused on quality improvement activities, interoperability, and cost. Those performance metrics, however, are not equity‐related and improving health equity between groups of beneficiaries would not result in additional payments unless the overall average increased as well. Even in disproportionate share hospitals and federally qualified health centers, which serve as a safety net for populations who experience inequities, financial programs are not explicitly aligned with achieving health equity, although they are implicitly tied to providers who care for groups at risk for inequities. Questions remain on the most effective approaches to incentive design and implementation of equity‐focused incentive programs.

#### 3.3.2 Research recommendation

A major component of AHRQ's Equity Agenda and Action Plan should be to commission the development of equity‐focused evidence‐based quality indicators in a process like AHRQ's Pediatric Quality Measurement Program which developed the Child Core Set of quality measures.^36^ A standardized health equity measure set would allow value‐based incentive programs to move away from the “rising tide floats all boats” strategy, which assumes improved performance for the entire population will improve health care for all at risk populations, and instead reward equity strategies that close performance gaps by meeting unique challenges faced by disadvantaged populations. Research should explore the relative effectiveness of types of incentives (e.g., bonuses, shared savings) and the relative effectiveness of incentive recipients (e.g., health care systems, senior executives, and front‐line workers). Results from this body of research should be used to develop toolkits to assist healthcare organizations with integrating equity metrics into their performance management systems.

Research is also needed on incentivizing quality and equity in resource limited health care delivery systems. Health care for minoritized populations is highly concentrated among a small group of health care providers^37^ and health care organizations that disproportionately serve diverse populations are more likely to deliver poor quality of care across the spectrum, from primary care to acute hospital care.^38^, ^39^, ^40^ Future, studies should measure the effect of tiered approaches to performance incentives that allow health care organizations with quality and equity challenges to be rewarded for incremental improvement not just benchmark achievement.

All incentive models require data for benchmarking. AHRQ can support access to disaggregated benchmarking data through interactive dashboards and query tools that leverage federal Medicare and Medicaid data and national survey data.

### 3.4 Theme 4: Health equity‐informed approaches to health system consolidation and access

#### 3.4.1 Rationale

US health care delivery systems are unevenly distributed geographically.^41^ This maldistribution can be traced to underinvestment in rural and inner‐city urban communities,^42^, ^43^, ^44^ discriminatory housing practices like redlining,^45^ and poor reimbursement by safety‐net insurance programs like Medicaid, especially in states that have not expanded Medicaid as part of the ACA.^46^ An estimated 80% of U.S. counties, which house more than one‐third of the population, have inadequate access to health care services and can be described as care deserts.^47^

Patients living in care deserts face delays in care that increase the likelihood of poor health outcomes^48^ and drive up the costs of care for patients, payors, and health care delivery systems, who bear the cost of uncompensated or undercompensated care for resulting serious illnesses.

Care deserts are expanding due to market‐driven consolidation of ownership of physician practices and hospitals into health care systems and large hospital/medical group conglomerates.^49^ The impact of consolidation on health equity is unclear. Consolidation has the potential to improve health care affordability and quality through centralization and standardization of administrative functions and scaling of evidence‐based guidelines, but there is limited evidence linking consolidation and structural integration to higher quality of care, patient care experiences, or lower spending.^50^ Some evidence indicates that consolidation may increase spending and reduce access to care for vulnerable populations.^51^, ^52^, ^53^ Even when reductions in costs and increases in efficiency occur, these savings are often not passed on to consumers through lower prices, with profits instead passed on to shareholders.^54^

#### 3.4.2 Recommendations

AHRQ supported research in health care delivery system consolidation and access should address how consolidations and closings are changing the geographic distribution of health care and the access inequities that are potentially created. Given that most consolidations involve multi‐state health systems acquiring smaller metro‐based systems,^49^ studies on consolidations should explore unintended impacts including loss of connection between health care organizations and local communities, reduction in autonomy of physicians to provide culturally tailored care to LEP and culturally diverse patients, and regionalization of specialty services that creates barriers to accessing in‐person care.

This work should include development of geographic information systems to track changes in the accessibility of health care for high need or special populations (e.g., Mediciad, children) and continued investment in national tracking systems for health care organizations and system consolidation and organizational capabilities, such as the Compendium of Health Systems^49^, ^55^ and the National Survey of Health care Organizations and Systems.^56^, ^57^ Further research should explore how equity should be considered in the decision to close emergency departments, outpatient facilities, and hospital locations as part of mergers and acquisitions and the effectiveness of delivery model innovations designed to reduce the impact of care deserts (e.g., telehealth, home monitoring).

### 3.5 Theme 5: Whole‐person health

#### 3.5.1 Rationale

The growing understanding that social conditions (i.e., nutrition, housing, transportation, environment, and other needs) impact population health has led to the adoption of screening, referral, and direct service models that leverage partnerships between health systems and community social service providers.^58^, ^59^ These partnerships mobilize resources and facilitate the creation of patient care coalitions, cross‐sector collaborations, and tailored interventions to address personal and environmental factors and maximize community health impact.^60^, ^61^, ^62^ Successful models for leveraging community partnerships and resources to address patient level social needs include:

- Employing community health workers (CHWs), who are frontline personnel with established trust and knowledge of community residents.^63^, ^64^ CHW encounters and interventions are associated with reduced emergency department visits, higher preventative care, and lower medical costs and incidence of disease.^65^, ^66^, ^67^, ^68^, ^69^
- Implementing health information exchanges (HIE), which allow for sharing of clinical, administrative, and social need data between health care providers who are not part of the same organizations.^70^, ^71^, ^72^, ^73^, ^74^
- Deploying provider organization resources to purchase social services from community organizations to addressing patients' unmet needs.^75^, ^76^, ^77^, ^78^, ^79^, ^80^

While promising, by focusing exclusively on individual needs, such strategies could redirect resources needed for community‐level solutions. They also can lead to a shift from horizontal integration, where health care organizations acquire or integrate with other providers that deliver similar services, to a vertically integrated structure, where organizations acquire or integrate with organizations offering different levels of care. This can displace community organizations and can lead to higher prices and spending.^81^, ^82^, ^83^

Health system approaches to health‐related social factors vary greatly.^84^, ^85^ Notably, hospitals that disproportionately care for more disadvantaged populations (including safety‐net hospitals, critical access hospitals, and rural hospitals) are not doing more, and in some cases doing much less, to address the social needs of their patients and their communities than others.^83^ These findings are concerning given that we would hope that these hospitals are engaged in significant efforts to address the unmet needs of their vulnerable patient populations. It is likely, however, that these hospitals' efforts are hampered by having limited financial resources, staffing constraints, weak financial incentives, and much more limited community resources and organizations with which to partner. State Medicaid programs have attempted to address these limitations by providing supplemental resources under federal waivers, with some success in California's recent whole‐person care pilot program.^86^

#### 3.5.2 Recommendations

Payer support for whole person care is still a developing field in need of further research.^87^ Research is needed on how public and private payors can enhance flexibilities in this space and on what systems (e.g., health care, community, faith‐based) are best suited to carry out this work. Another important question is what innovative solutions can be employed by communities and health care systems with limited infrastructure and capacity for engagement, even when payment models are available. In addition, to understand the effectiveness of whole‐person care, we must develop means of measurement that cross silos of health care and social support. AHRQ is well‐positioned to support research activities that engage other federal entities responsible for addressing social risk factors such as the Environmental Protection Agency, the Department of Transportation, and the Department of Housing and Urban Development. Co‐sponsoring research with these agencies can help promote enhanced surveillance of agency interventions and study the health and equity effects of these policies and programs that impact social risk factors. AHRQ can also support the development of secondary linked data sets available through census data centers to promote and accelerate research on health outcomes linked to social needs (i.e., the previous use of linked employer firms to household MEPS in MEPS‐IC and MEPS‐HC from 1996, the HUD‐NHIS linkage used by NCHS, etc.).

### 3.6 Theme 6: Whole community investment

#### 3.6.1 Rationale

Health care delivery systems have the potential to extend beyond individual patient care and play a significant role in addressing health and health care inequities in the communities they serve, which are often under‐resourced, de facto segregated, and facing some of the starkest inequities in health care insurance coverage, access, and clinical outcomes due to racially targeted disinvestments. Key in these efforts is meaningful engagement, investment, and partnerships with the local communities that health care systems serve. Nonprofit hospital status exempts health systems from paying federal and state taxes if they demonstrate that they are providing a benefit to their communities. While community benefit spending traditionally meant charity care, the types of services hospitals can provide to document investment in their communities has broadened over time.^88^ Today, community benefit spending, in the form of direct spending on community health and broad community health initiatives, has the potential to be a powerful tool for addressing the burden of the social determinants of health within communities. However, this promise may be blunted by limited oversight^89^ and the growth of for‐profit hospitals, who are not incentivized by tax exemption.^90^, ^91^

In their role as anchor institutions in their communities health care delivery system can engage in “groundwater” strategies.^92^, ^93^ The term groundwater is derived from a structural racism metaphor. Simply stated, when we notice a stream full of dead fish, the problem is likely to be in the water not in each individual fish. Examples of “Groundwater” strategies include:

- Supporting poverty reduction and access to health insurance in their communities by hiring locally and providing livable wages and career development opportunities for community members, who may not otherwise have a viable pathway to pursue a health care‐related career.^92^
- Financing and leveraging resources to support local communities' efforts into building green spaces, public transportation, affordable housing and childcare options, and local grocery stores.^94^, ^95^
- Leveraging their role in their communities to inform policy conversations related to the social determinants of health, helping policymakers make the connection between policy decisions that may seem unrelated to health but are inextricably linked to health outcomes and people's ability to live and thrive (e.g., affordable housing, transportation).^92^, ^96^
- Being active voices in social issues, including racism and other biases, that affect the health and well‐being of the population.

#### 3.6.2 Recommendations

Though these strategies have face validity and have shown promising health‐related effects in modestly size implementations, additional research is needed on which approaches have the greatest impact on which outcomes and on best practices for implementation and scale. This will require supporting innovative health services research studies examining outcomes at the intersections of race, racism, economic adversity, and other identities. Academic health care systems can, and should, partner with communities to conduct such research. To facilitate such partnerships, AHRQ should develop new health equity research funding models with funding allocated directly to community organizations working in partnership with universities or hospitals, like the Small Business Innovation Research (SBIR) model.

### 3.7 Cross cutting research recommendations

Two potential roadblocks to successful implementation of this agenda are time and people. A well‐trained research workforce, with long term funding opportunities, is necessary to study the effects of interventions rooted in long term inequities. We therefore add two more cross‐cutting research recommendation: (1) Expansion of R25, T32, K series, and R01 diversity supplement mechanisms to train the next generation of health services researchers in methodologically rigorous health equity research and (2) development of funding mechanisms that go beyond the 5‐year window of a traditional R01 award.

## 4 DISCUSSION

Using stakeholder input from the AHRQ Health Equity Summit, a targeted review of the literature, and a consensus of experts, we have identified six priority themes for research on health care systems to promote health equity and population health. This list is by no means exhaustive, but it offers myriad research‐ready opportunities to learn what works and what does not. The research and action recommendations reflect the depth and breadth of the challenges of unremitting health care inequities, ranging from workforce development, to building of national data repositories, to studying the impact of a diverse set of policy and practice interventions at the health care system, community, and national levels. Taken together, however, they form an agenda with the potential to build the evidence‐base needed to re‐form the US health care delivery system to one that supports everyone's opportunity to attain optimal health.

We note that we intentionally did not focus on the role of hospital quality and safety programs as drivers of health equity improvement. These programs, while important, tend to focus on practice and outcomes in circumscribed patient populations. There have been meaningful improvements in discrete measures of health care quality over the past two decades. The 2022 AHRQ National Healthcare Quality and Disparities Report documents notable overall national improvements in health care delivery for conditions including breast cancer and HIV/AIDS, but persistent health care inequities for minoritized racial and ethnic populations and those experiencing socioeconomic disadvantage remained.^97^ This dissonance illustrates that improvement for discrete care measures, while laudable, do not automatically lead to reduced health care inequities and are insufficient to substantially advance health equity.

We note a few limitations in this work. First, this study defines health care delivery organizations as entities that deliver health services directly to patients. This can range from small community hospitals to multi‐state integrated delivery systems. All content may not apply to all organization types. Second, our presentation of themes does not fully address all populations that experience disparities (e.g., LGBTQ+, disability) nor address unique equity challenges within subpopulations (e.g., women, children). These are important issues that demand further exploration. Finally, we do not discuss the precarious political environment that pushes back against research on health equity under the dubious rationale that such work is “divisive.” For AHRQ, building a comprehensive health equity research and action plan may at times be an uphill battle but it is a battle worth fighting.

## 5 CONCLUSION

AHRQ has the potential to become the home for research that demonstrates the impact of health care delivery systems on health equity, identifies effective care delivery models, and promotes the dissemination of best practices to enable population‐level equity improvements. A comprehensive portfolio will require dedicated funding for investigator‐initiated implementation and outcomes research, research on innovative models of clinical practice and community engagement, and equity‐focused health services and policy research training programs to diversify the investigator pipeline.

## FUNDING INFORMATION

This study was prepared with financial support from the Agency for Health care Research and Quality.

## CONFLICT OF INTEREST STATEMENT

None.

## Supporting information


**Data S1.** Supporting Information
